# The Pathology and Treatment of Sexual Impotence

**Published:** 1899-07

**Authors:** 


THE PATHOLOGY AND TREATMENT OF SEXUAL IMPOTENCE. By VICTOR G. VECKI, M. D. From the Author's Second German Edition, revised and rewritten. Duodecimo, pp. 291. Philadelphia: W. B. Saunders. 1899.

The second edition of Vecki's work finds the profession somewhat changed in its attitude upon this subject. The medieval way of treating this subject by not treating of it at all, is no longer tenable in these latter days of the XIXth century and the profession owes a debt of gratitude to Vecki for fighting manfully against a storm of opposition. Casper, Eulenburg, Fürbringer, Krafft-Ebing, Schrenck, Notzing and others have given Vecki's work due consideration and the work is destined to do a great deal of good. The author's ideas and suggestions on treatment are sound and embrace the latest additions to our knowledge of the therapeutics on this subject. The different forms of impotence are carefully considered and well depicted. The book is well printed, happily has no illustrations, and is modest and unobtrusive in its appearance. W. C. K.

